# Draft genome sequence of *Acinetobacter baumannii* Ab10, a clinical isolate from Hospital University Sains Malaysia (HUSM), Malaysia

**DOI:** 10.1128/mra.01084-23

**Published:** 2024-03-19

**Authors:** Nik Zakuan Hakim Nik Mohd Nazri, Kirnpal Kaur Banga Singh, Mohamad Izwan Ismail

**Affiliations:** 1 Fakulti Sains Gunaan, UiTM Cawangan Sarawak Kampus Mukah, Mukah, Sarawak, Malaysia; 2 Department of Medical Microbiology and Parasitology, School of Medical Sciences, Universiti Sains Malaysia, Health Campus, Kubang Kerian, Kelantan, Malaysia; Department of Biological Sciences, Wellesley College, Wellesley, Massachusetts, USA

**Keywords:** *Acinetobacter baumannii*, whole genome, sequencing, antimicrobial, resistance

## Abstract

*Acinetobacter baumannii* strain Ab10 retrieved in Malaysia in 2017 represents a pathogen carrying multiple antibiotic-resistant genes (*blaOXA-23*, *ant(3”)-Ila*, *blaADC-32*, and *blaOXA-699*). We introduce the 3.89 Mbp genome sequence from short-read sequencing (Illumina’s NovaSeq6000).

## ANNOUNCEMENT

An *Acinetobacter baumannii* strain Ab10 was recovered from the peripheral blood of a Malay female pediatric patient (1 year 5 months) in June 2017 at Hospital Universiti Sains Malaysia (HUSM) Kubang Kerian, Kelantan, Malaysia. The patient presented with high-grade fever associated with vesicular rash secondary to *Varicella zoster* infection with toxic shock syndrome and was ventilated for 8 days. Blood cultures were performed using BD BACTEC FX Blood Culture System, and species identification was done using standard culture procedure and Vitek 2 Identification System (BioMérieux). Isolates were grown in tryptic soy broth at 37°C overnight and stored in 15% glycerol. The sample was retrieved from storage (−80°C) and grown in blood agar at 37°C for 18 hours. The sample collection was in compliance with the Universiti Sains Malaysia Human Ethical Committee (JEPeM, USM) (approval code USM/JEPeM/18010015).

Whole-cell DNA was processed from a culture grown on Mueller Hinton Agar for 18 hours at 37°C. Gram stain was used to check general cell morphology. The DNA was sent to a third-party company (GeneSeq) for whole-genome sequencing. DNA extraction was performed using the WizPrep gDNA mini kit (WizBio, South Korea) per the manufacturer’s instructions. Illumina library preparation was done using the Covaris UltraSonicator (Covaris, Woburn, USA) and processed using the NEB Ultra II Library Preparation kit (NEB, Ipswich, USA). The library was quantified using the Qubit fluorometer (Invitrogen, Waltham, USA) and TapeStation (Agilent, Santa Clara, USA), followed by concentration adjustment and sequencing on a NovaSeq6000 (150 bp read length, paired-end). A total of 10,148,656 reads were acquired and quality-controlled using Galaxy’s FastQC v.0.74+galaxy0 (1). The raw reads were adapter-trimmed and quality-trimmed with fastp v.0.23 (2) and assembled using Unicycler v.0.4.8, retaining only contigs of 500 bp or longer (3). Genome assembly statistics were obtained using QUAST v.5.2.0, reporting 144 contigs with the largest contig at 227,605 bp. The strain exhibited a total genome length of 3,890,005 bp at 196× coverage, with a 39.1% GC content, and an N50 of 76,135 (4). Genome completeness was evaluated using BUSCO5 v.5.3 with a complete single-copy gene value of 95.2% (5).

The NCBI PGAP annotation v.6.3 (JAPDKJ000000000) identified 3,849 genes, 3,651 protein-coding genes, and 71 RNAs. The nearest neighbor organism was determined to be *A. baumannii* ATCC 17978 according to RAST v.2.0 (6–8) and ANI Calculator with an OrthoANIu value of 97.6% (9, 10). Multilocus Sequence Typing (MLST) analysis revealed Ab10 to be of sequence type 1904 (Pasteur scheme) and unknown sequence type (Oxford scheme) (11). Gene analysis using Abricate v.1.0.1 (https://github.com/tseemann/abricate) (12) suggested potential resistance to carbapenem, spectinomycin, streptomycin, cephalosporin, and beta-lactam (13–15). In addition, one integrated prophage genome of 41.6 kbp (bases 2943-44619 region position) was detected using the PHASTER (upgrade 6) (16, 17). Insertion sequences ISAba26, ISAba63, ISAba22, ISAba1, and ISAba19 were detected using ISfinder (https://www-is.biotoul.fr/) (18). Default parameters were used for all software unless otherwise noted.

## Data Availability

The draft whole-genome sequence of *A. baumannii* Ab10 is available through NCBI under BioProject accession number PRJNA881790, Biosample ID SAMN30915916, SRA accession number SRR26679157, and genome accession number JAPDKJ000000000.

## References

[1] Andrew S. 2010. Internet. FastQC: a quality control tool for high throughput sequence data. Available from: https://www.bioinformatics.babraham.ac.uk/projects/fastqc/

[2] Chen S, Zhou Y, Chen Y, Gu J. 2018. Fastp: an ultra-fast all-in-one FASTQ preprocessor. Bioinformatics 34:i884–i890. doi:10.1093/bioinformatics/bty560 30423086 PMC6129281

[3] Wick RR, Judd LM, Gorrie CL, Holt KE. 2017. Unicycler: resolving bacterial genome assemblies from short and long sequencing reads. PLoS Comput Biol 13:e1005595. doi:10.1371/journal.pcbi.1005595 28594827 PMC5481147

[4] Gurevich A, Saveliev V, Vyahhi N, Tesler G. 2013. QUAST: quality assessment tool for genome assemblies. Bioinformatics 29:1072–1075. doi:10.1093/bioinformatics/btt086 23422339 PMC3624806

[5] Simão FA, Waterhouse RM, Ioannidis P, Kriventseva EV, Zdobnov EM. 2015. BUSCO: assessing genome assembly and annotation completeness with single-copy orthologs. Bioinformatics 31:3210–3212. doi:10.1093/bioinformatics/btv351 26059717

[6] Aziz RK, Bartels D, Best AA, DeJongh M, Disz T, Edwards RA, Formsma K, Gerdes S, Glass EM, Kubal M, et al.. 2008. The RAST server: rapid annotations using subsystems technology. BMC Genomics 9:75. doi:10.1186/1471-2164-9-75 18261238 PMC2265698

[7] Brettin T, Davis JJ, Disz T, Edwards RA, Gerdes S, Olsen GJ, Olson R, Overbeek R, Parrello B, Pusch GD, Shukla M, Thomason JA 3rd, Stevens R, Vonstein V, Wattam AR, Xia F. 2015. RASTtk: a modular and extensible implementation of the RAST algorithm for building custom annotation pipelines and annotating batches of genomes. Sci Rep 5:8365. doi:10.1038/srep08365 25666585 PMC4322359

[8] Overbeek R, Olson R, Pusch GD, Olsen GJ, Davis JJ, Disz T, Edwards RA, Gerdes S, Parrello B, Shukla M, Vonstein V, Wattam AR, Xia F, Stevens R. 2014. The SEED and the rapid annotation of microbial genomes using subsystems technology (RAST). Nucleic Acids Res 42:D206–14. doi:10.1093/nar/gkt1226 24293654 PMC3965101

[9] BioCloud EZ. 2023 ANI calculator (pairwise). Available from: https://www.ezbiocloud.net/tools/ani

[10] Yoon SH, Ha SM, Lim J, Kwon S, Chun J. 2017. A large-scale evaluation of algorithms to calculate average nucleotide identity. Antonie Van Leeuwenhoek 110:1281–1286. doi:10.1007/s10482-017-0844-4 28204908

[11] Larsen MV, Cosentino S, Rasmussen S, Friis C, Hasman H, Marvig RL, Jelsbak L, Sicheritz-Pontén T, Ussery DW, Aarestrup FM, Lund O. 2012. Multilocus sequence typing of total-genome-sequenced bacteria. J Clin Microbiol 50:1355–1361. doi:10.1128/JCM.06094-11 22238442 PMC3318499

[12] Seeman T. 2019 Abricate. Internet. Available from: https://github.com/tseemann/abricate

[13] Zhang G, Leclercq SO, Tian J, Wang C, Yahara K, Ai G, Liu S, Feng J. 2017. A new subclass of intrinsic aminoglycoside nucleotidyltransferases, ANT(3")-II, is horizontally transferred among Acinetobacter spp. by homologous recombination. PLoS Genet 13:e1006602. doi:10.1371/journal.pgen.1006602 28152054 PMC5313234

[14] Hou C, Yang F. Drug-resistant gene of blaOXA-23, blaOXA-24, blaOXA-51 and blaOXA-58 in Acinetobacter baumannii. Int J Clin Exp Med. 2015;8(8):13859–63. 26550338 PMC4613023

[15] Wareth G, Brandt C, Sprague LD, Neubauer H, Pletz MW. 2021. WGS based analysis of acquired antimicrobial resistance in human and non-human Acinetobacter baumannii isolates from a German perspective. BMC Microbiol 21:210. doi:10.1186/s12866-021-02270-7 34243717 PMC8272256

[16] Arndt D, Grant JR, Marcu A, Sajed T, Pon A, Liang Y, Wishart DS. 2016. PHASTER: a better, faster version of the PHAST phage search tool. Nucleic Acids Res 44:W16–W21. doi:10.1093/nar/gkw387 27141966 PMC4987931

[17] Zhou Y, Liang Y, Lynch KH, Dennis JJ, Wishart DS. 2011. PHAST: a fast phage search tool. Nucleic Acids Res 39:W347–52. doi:10.1093/nar/gkr485 21672955 PMC3125810

[18] Siguier P, Perochon J, Lestrade L, Mahillon J, Chandler M. 2006. ISfinder: the reference centre for bacterial insertion sequences. Nucleic Acids Res 34:D32–6. doi:10.1093/nar/gkj014 16381877 PMC1347377

